# Supplementary material for: Natural silencing of quorum-sensing activity protects Vibrio parahaemolyticus from lysis by an autoinducer-detecting phage
Source: PLoS Genet. 2023 Jul 31;19(7):e1010809. doi: 10.1371/journal.pgen.1010809 (PMC10426928; doi:10.1371/journal.pgen.1010809)
Supplement: S1 Table — (DOCX) [file pgen.1010809.s001.docx]

**Table S1 Numerical values, and associated p-values, for heatmaps in Figure 4A.**

|  | ***vqmR-vqmA_882_^+^*** | | ***luxO^+^*** | | ***vqmR-vqmA_882_^+^ luxO^+^*** | |
| --- | --- | --- | --- | --- | --- | --- |
| VP882 gene | log_2_FC | P-value | log_2_FC | P-value | log_2_FC | P-value |
| *gp01* | 0.868 | 8.52E-02 | 3.282 | 3.76E-10 | 3.368 | 1.86E-10 |
| *gp04* | 0.467 | 2.85E-03 | 2.778 | 1.35E-20 | 3.835 | 5.68E-25 |
| *gp05* | 0.351 | 1.88E-02 | 2.648 | 3.47E-20 | 3.402 | 1.37E-23 |
| *gp06* | 0.460 | 7.41E-04 | 2.499 | 1.68E-21 | 3.147 | 9.66E-25 |
| *gp07* | 0.551 | 3.42E-04 | 2.643 | 1.59E-20 | 3.352 | 8.66E-24 |
| *gp10* | 0.539 | 8.49E-05 | 2.842 | 1.08E-23 | 3.399 | 3.33E-26 |
| *gp11* | 0.529 | 2.87E-04 | 2.758 | 5.01E-22 | 3.452 | 3.86E-25 |
| *gp14* | 0.490 | 5.77E-03 | 2.243 | 6.61E-15 | 2.920 | 5.26E-18 |
| *gp16* | 0.443 | 2.37E-02 | 2.061 | 1.16E-14 | 2.664 | 1.59E-18 |
| *gp17* | 0.378 | 6.54E-03 | 2.149 | 3.81E-19 | 2.794 | 5.86E-23 |
| *gp18* | 0.440 | 1.03E-03 | 2.007 | 6.81E-19 | 2.714 | 2.85E-23 |
| *gp19* | 0.541 | 5.06E-04 | 2.230 | 6.66E-18 | 2.897 | 1.82E-21 |
| *gp20* | 0.532 | 1.72E-04 | 2.216 | 1.41E-19 | 2.812 | 6.13E-23 |
| *gp21* | 0.499 | 1.03E-03 | 2.396 | 3.12E-19 | 2.913 | 6.09E-22 |
| *gp22* | 0.497 | 1.84E-04 | 2.384 | 1.40E-21 | 2.980 | 9.09E-25 |
| *gp23* | 0.643 | 1.13E-04 | 2.371 | 1.07E-17 | 3.099 | 3.66E-21 |
| *gp24* | 0.395 | 3.98E-03 | 2.223 | 1.81E-19 | 2.750 | 1.85E-22 |
| *gp25* | 0.321 | 7.45E-03 | 2.382 | 2.36E-22 | 2.490 | 5.45E-23 |
| *gp26* | 0.415 | 2.23E-02 | 2.260 | 5.52E-14 | 2.447 | 7.62E-15 |
| *gp27* | 0.516 | 1.38E-03 | 2.443 | 5.13E-18 | 2.695 | 2.77E-19 |
| *gp28* | 0.552 | 1.51E-03 | 2.537 | 1.42E-17 | 3.152 | 2.19E-20 |
| *gp29* | 0.595 | 4.68E-05 | 2.842 | 9.15E-23 | 3.306 | 7.26E-25 |
| *gp30* | 0.489 | 1.67E-03 | 2.106 | 7.77E-17 | 2.614 | 1.01E-19 |
| *gp31* | 0.576 | 4.80E-04 | 2.078 | 3.43E-16 | 2.907 | 1.03E-20 |
| *gp32* | 0.478 | 2.90E-04 | 1.924 | 1.36E-18 | 2.676 | 2.58E-23 |
| *gp33* | 0.277 | 2.68E-01 | 1.183 | 9.93E-06 | 2.155 | 5.05E-12 |
| *gp35* | 0.478 | 3.74E-04 | 1.832 | 1.38E-17 | 2.321 | 6.13E-21 |
| *gp36* | 0.317 | 1.87E-01 | 1.510 | 2.31E-07 | 1.890 | 2.59E-09 |
| *gp37* | 0.413 | 4.50E-04 | 2.461 | 1.01E-23 | 2.679 | 5.96E-25 |
| *gp38* | 0.283 | 1.89E-02 | 2.371 | 3.94E-22 | 2.615 | 1.54E-23 |
| *gp39* | 0.385 | 6.27E-03 | 2.098 | 3.00E-18 | 2.977 | 3.66E-23 |
| *gp42* | 0.457 | 1.11E-01 | 1.842 | 8.21E-09 | 2.012 | 8.44E-10 |
| *gp45* | 0.018 | 8.93E-01 | -0.344 | 1.19E-02 | -0.231 | 8.44E-02 |
| *gp46* | 0.224 | 3.05E-02 | -0.587 | 7.95E-07 | -0.532 | 4.62E-06 |
| *gp47* | -0.119 | 3.19E-01 | -0.702 | 7.74E-07 | -0.865 | 1.20E-08 |
| *gp48* | 0.134 | 4.25E-01 | -0.450 | 1.27E-02 | -0.525 | 4.51E-03 |
| *gp49* | 0.203 | 1.75E-01 | -0.670 | 9.08E-05 | -0.166 | 2.79E-01 |
| *gp51* | 0.340 | 6.25E-01 | -1.221 | 1.90E-01 | 0.543 | 4.53E-01 |
| *gp52* | 0.149 | 2.48E-01 | 0.599 | 2.95E-05 | 0.794 | 2.19E-07 |
| *parA* | 0.247 | 4.25E-02 | 0.804 | 3.83E-08 | 0.828 | 2.03E-08 |
| *telN* | 0.481 | 2.51E-04 | 0.019 | 8.76E-01 | 0.183 | 1.34E-01 |
| *qtip* | 0.156 | 5.66E-01 | 0.467 | 9.03E-02 | 0.705 | 1.19E-02 |
| *vqmA_Phage_* | 0.066 | 6.48E-01 | 1.277 | 5.71E-11 | 1.796 | 3.29E-15 |
| *repA* | 0.272 | 1.77E-02 | 1.757 | 1.51E-18 | 2.179 | 1.23E-21 |
| *cI* | -0.040 | 7.25E-01 | -0.159 | 1.68E-01 | 0.180 | 1.19E-01 |
| putative *cro* | 0.210 | 9.26E-02 | 1.668 | 1.74E-16 | 2.110 | 8.64E-20 |
| putative *dskA* | 0.528 | 8.35E-04 | 1.861 | 4.85E-16 | 2.093 | 1.03E-17 |
| *q* | 0.159 | 2.18E-01 | 1.485 | 1.04E-14 | 1.738 | 6.95E-17 |
| unknown | 0.148 | 2.89E-01 | 0.092 | 5.12E-01 | 0.424 | 3.98E-03 |
| *gp69* lysin | 0.234 | 6.33E-02 | 2.465 | 8.39E-22 | 3.091 | 5.03E-25 |
| *gp70* lysin | 0.336 | 5.75E-03 | 2.686 | 6.14E-24 | 3.146 | 3.45E-26 |
| *gp71* lysin | 0.207 | 9.20E-02 | 2.305 | 4.35E-21 | 2.723 | 1.83E-23 |
