# Supplementary material for: Natural silencing of quorum-sensing activity protects Vibrio parahaemolyticus from lysis by an autoinducer-detecting phage
Source: PLoS Genet. 2023 Jul 31;19(7):e1010809. doi: 10.1371/journal.pgen.1010809 (PMC10426928; doi:10.1371/journal.pgen.1010809)
Supplement: S2 Table — (DOCX) [file pgen.1010809.s002.docx]

##### Table S2 Strains used in this study.

| **Strain** | **Genotype** | **Reference** |
| --- | --- | --- |
| *V. cholerae* str. C6706 | *vqmA_Vc_::vqmA_Vc_-3XFLAG* | [7] |
|  | P*vqmA_RIMD_-vqmA_Vc_-3XFLAG* | This study |
| *E. coli* BW25113 | *lacIq*, *rrnBT14*, Δ*lacZWJ16*, *hsdR514*, Δ*araBADAH33*, Δ*rhaBADLD78,* Δ*tdh* | [8] |
| *E. coli* S17λ*pir* | Δ*lacU169 (ΦlacZ*Δ*M15), recA1, endA1, hsdR17, thi-1, gyrA96, relA1, λpir* | [34] |
| *E. coli* TOP10 | F- *mcrA* Δ(*mrr*-*hsdRMS*-*mcrBC*) Φ80*lacZ*Δ*M15* Δ*lacX74 recA1 araD139* Δ(*ara leu*)*7697* *galU galK rpsL* (Str^R^) *endA1 nupG* | Invitrogen |
| *V. parahaemolyticus* O3:K6 RIMD2210633 | *vqmA_RIMD_*::*vqmA_RIMD_*-*3XFLAG* | This study |
|  | P*vqmA_Vc_*-*vqmA_RIMD_*-*3XFLAG* | This study |
| *V. parahaemolyticus* O3:K6 strain 882 | *vqmA_882_::vqmA_882_*-*3XFLAG* (882 parent) | This study |
|  | P*vqmA_882_-vqmA_882_::vqmA_882_*-*3XFLAG* *(vqmA_882_^+^*) | This study |
|  | *vqmR^+^-*P*vqmA_882_-vqmA_882_::vqmA_882_*-*3XFLAG* *(vqmR-vqmA_882_^+^*) | This study |
|  | *vqmA_882_::vqmA_882_*-*3XFLAG; luxO_882_::luxO_RIMD_* (*luxO^+^*) | This study |
|  | *vqmR^+^-*P*vqmA_882_-vqmA_882_::vqmA_882_*-*3XFLAG; luxO_882:_:luxO_RIMD_* (*vqmR-vqmA_882_^+^ luxO^+^*) | This study |
|  | *vqmA_882_::vqmA_882_*-*3XFLAG; luxO_882_::luxO^D61E^* (*luxO^D61E^*) | This study |
|  | *vqmA_882_::vqmA_882_*-*3XFLAG; luxO_882_::luxO^D61A^* (*luxO^D61A^*) | This study |
|  | *vqmA_882_::vqmA_882_*-*3XFLAG; luxO_882_::luxO_882_^D61E^* (*luxO_882_^D61E^*) | This study |
|  | *vqmA_882_::vqmA_882_*-*3XFLAG; luxO_882_::luxO_882_^D61A^* (*luxO_882_^D61A^*) | This study |
|  | *vqmA_882_::vqmA_882_*-*3XFLAG; luxO_882_::luxO_882_* (*luxO-3XFLAG*) | This study |
|  | *vqmA_882_::vqmA_882_*-*3XFLAG; luxO_882_::luxO_RIMD_-3XFLAG* (*luxO^+^-3XFLAG*) | This study |
