# Supplementary material for: Natural silencing of quorum-sensing activity protects Vibrio parahaemolyticus from lysis by an autoinducer-detecting phage
Source: PLoS Genet. 2023 Jul 31;19(7):e1010809. doi: 10.1371/journal.pgen.1010809 (PMC10426928; doi:10.1371/journal.pgen.1010809)
Supplement: S3 Table — (DOCX) [file pgen.1010809.s003.docx]

##### Table S3 Primers and gBlocks used in this study.

| **Name** | **Sequence (5 - 3)** | **Purpose; Template** |
| --- | --- | --- |
| ODO-786 | CTGCTGACTCTGATTGTGCTG | qPCR; phage VP882 *gp69* |
| ODO-787 | TCGTGAGAGGTGATGTACTTCTC | qPCR; phage VP882 *gp69* |
| ODO-30 | CTAAGGGGCAATCTCTACAAGACCCA | qPCR; strain 882 *hfq* |
| ODO-370 | ACCATTTGGTTTACAGTATTTTTCAGCAG | qPCR; strain 882 *hfq* |
| ODO-943 | TTCAAACAGATCCTGAAGCGCCTC | First-round amplification VP882 *qtip* 3Kb upstream flanking sequence; from strain 882 gDNA |
| ODO-944 | TACTGCGATGAGTGGCAGGGCGGGGCGTAAGATTAGTCATCGAGTGCCTTTTGGCTG | First-round amplification VP882 *qtip* 3Kb upstream flanking sequence; from strain 882 gDNA |
| ODO-947 | GGCGTGGTCCGCCCGAGGGCAGAGCCATGACACACGAATACACTCCTTGTAAGTGATTGT | First-round amplification VP882 *qtip* 3Kb downstream flanking sequence; from strain 882 gDNA |
| ODO-948 | TCAACTACATCCGCCTCGAGGG | First-round amplification VP882 *qtip* 3Kb downstream flanking sequence; from strain 882 gDNA |
| ODO-945 | TTACGCCCCGCCCTGCCACTCA | First-round amplification of *cat-traJ*; Ec-OD603 |
| ODO-946 | TCATGGCTCTGCCCTCGGGCGGAC | First-round amplification of *cat-traJ*; Ec-OD603 |
| ODO-949 | AATTGGCAGCTCGTACCCTTC | Second round amplification to construct *qtip*::*cm*; ODO-943x944, ODO-947x948, ODO-9454x946 |
| ODO-950 | GACACTCTTTGCATCGATGAGGCC | Second round amplification to construct *qtip*::*cm*; ODO-943x944, ODO-947x948, ODO-9454x946 |
| ODO-951 | TCATGGCTCTCAAGACTTTTTGATCA | Intromolecular reclosure to delete Δ91-102 *luxO;* FJS-S113 and FJS-S114 |
| ODO-952 | GCCGTCATGAAAATGATCGGCA | Intromolecular reclosure to delete Δ91-102 *luxO;* FJS-S113 and FJS-S114 |
| pRE112-1 | ATGCAGTTCACTTACACCGCTTC | Amplification of pRE112 backbone; pRE112 plasmid |
| pRE112-2 | GGGATCGGGCCCTATCACTT | Amplification of pRE112 backbone; pRE112 plasmid |
| ODO-651 | GAAGCGGTGTAAGTGAACTGCATATCAGTTCGATGATGTTCGAATACCGC | Amplification to construct *luxO_RIMD_* in pRE112; from *V. parahaemolyticus* RIMD2210633 gDNA |
| ODO-656 | AAGTGATAGGGCCCGATCCCTGTTACGACTCTGACCAAACGAATGG | Amplification to construct *luxO_RIMD_* in pRE112; from *V. parahaemolyticus* RIMD2210633 gDNA |
| ODO-619 | GTGAGTCCTCTTGTGAATATTCCAGTCAA | Amplification of pRE112 backbone containing *vqmA_RIMD_-3XFLAG*; pRE112-*vqmA_RIMD_-3XFLAG* |
| ODO-620 | GGATCAAAAAAAAAGCCAGCCTGAAGA | Amplification of pRE112 backbone containing *vqmA_RIMD_-3XFLAG*; pRE112-*vqmA_RIMD_-3XFLAG* |
| ODO-621 | TCTTCAGGCTGGCTTTTTTTTTGATCCAAACAGTCCCCGAATAGGCAA | Amplification of *vqmA_Vc_* promoter to construct *PvqmA_Vc_-vqmA_RIMD_-3XFLAG*; from *V. cholerae* gDNA |
| ODO-622 | TTGACTGGAATATTCACAAGAGGACTCACGCCATATCCTCCACTGGAAATGCG | Amplification of *vqmA_Vc_* promoter to construct *PvqmA_Vc_-vqmA_RIMD_-3XFLAG*; from *V. cholerae* gDNA |
| ODO-626 | GCTTATGCCATTGTTTTCATTTTTGAGATAGAAAAAAAGCCAGCCTGAAGACGG | Amplification of pKAS backbone containing *vqmA_Vc_-3XFLAG*; pKAS-*vqmA_Vc_-3XFLAG* (pKP485) |
| ODO-629 | TGGGGCCAAATAGCAGTAGTGAGTCCTCTTGTGCCTAACCATCTGACATTAG | Amplification of pKAS backbone containing *vqmA_Vc_-3XFLAG*; pKAS-*vqmA_Vc_-3XFLAG* (pKP485) |
| ODO-627 | CCGTCTTCAGGCTGGCTTTTTTTCTATCTCAAAAATGAAAACAATGGCATAAGC | Amplification of *vqmA_RIMD_* promoter to construct P*vqmA_RIMD_-vqmA_Vc_-3XFLAG*; from *V. parahaemolyticus* RIMD2210633 gDNA |
| ODO-912 | TACTGCTATTTGGCCCCAAGCC | Amplification of *vqmA_RIMD_* promoter to construct P*vqmA_RIMD_-vqmA_Vc_-3XFLAG*; from *V. parahaemolyticus* RIMD2210633 gDNA |
| ODO-427 | TTAATTAACTCGAGCGGTACCCGCCG | Amplification of the pEVS backbone containing luciferase genes; P*vqmR_Vc_*-lux plasmid (EcOD119) |
| ODO-426 | ATGACTAAAAAAATTTCATTCATTATTAACGGCCAGG | Amplification of the pEVS backbone containing luciferase genes; P*vqmR_Vc_*-lux plasmid (EcOD119) |
| ODO-860 | CGGCGGGTACCGCTCGAGTTAATTAAATGTGAGATTAACTTACGGTCAATGTACAA | Amplification of *vqmR_RIMD_* promoter to insert into pEVS-*lux;* from *V. parahaemolyticus* RIMD2210633 gDNA |
| ODO-558 | CCTGGCCGTTAATAATGAATGAAATTTTTTTAGTCATCAGGGTCGTATGCATCCTAGAGC | Amplification of *vqmR_RIMD_* promoter to insert into pEVS-*lux;* from *V. parahaemolyticus* RIMD2210633 gDNA |
| ODO-930 | CGGCGGGTACCGCTCGAGTTAATTAAAGCATCATCCCCTTCGCTTATTGA | Amplification of *qtip* promoter to insert into pEVS-*lux*; from strain 882 gDNA |
| ODO-931 | CCTGGCCGTTAATAATGAATGAAATTTTT | Amplification of *qtip* promoter to insert into pEVS-*lux*; from strain 882 gDNA |
| ODO-551 | GGGTTGAGAAGCGGTGTAAGTGAACTGCATAATCACAATCTGCGTGTAAGCATGATCA | Amplification of *vqmA_RIMD_* upstream sequence to construct *vqmA_RIMD_*-*3XFLAG*; from *V. parahaemolyticus* RIMD2210633 gDNA |
| ODO-552 | ACGCCTGAATAAGTGATAGGGCCCGATCCCTCACGTTACTTCTCGTCGCTTCATTCT | Amplification of *vqmA_RIMD_ and vqmA_882_* downstream sequence to construct *vqmA_RIMD_* - and *vqmA_882_ -3XFLAG*; from strain 882 gDNA |
| ODO-553 | GGGTTGAGAAGCGGTGTAAGTGAACTGCATCACAACAAAATGTTAGGAATATCAATAAGA | Amplification of *vqmA_882_* upstream sequence to construct *vqmA_882_*-*3XFLAG* in pRE112; from strain 882 gDNA |
| ODO-586 | TACTGCTATTTGGCCCCAAGCCATC | Amplification of *vqmA_RIMD_ and vqmA_882_* upstream sequence to construct *vqmA_RIMD_* - and *vqmA_882_ -3XFLAG*; from strain 882 and *V. parahaemolyticus* RIMD2210633 gDNA |
| ODO-529 | CGCCACGCCGCTTTATCTTTATAAACA | Amplification of *vqmA_RIMD_ and vqmA_882_* downstream sequence to construct *vqmA_RIMD_* - and *vqmA_882_ -3XFLAG*; from *V. parahaemolyticus* RIMD2210633 gDNA |
| ODO-1 | GCCATATCCTCCACTGGAAATGC | Amplification of pBR322-pBAD backbone; pBAD-*vqmA_Vc_-3XFLAG* (BB-Ec0042) |
| ODO-2 | TAAGCAACAACGTCAAGCTGATTG | Amplification of pBR322-pBAD backbone; pBAD-*vqmA_Vc_-3XFLAG* (BB-Ec0042) |
| ODO-635 | GCATTTCCAGTGGAGGATATGGCGTGAGTCCTCTTGTGAATATTCCAG | Amplification of *vqmA_RIMD_* to insert into pBR322-pBAD; *vqmA_RIMD_-3XFLAG* gBlock |
| ODO-636 | CAATCAGCTTGACGTTGTTGCTTATTATTTATCGTCATCTTTGTAGTCGATATCATG | Amplification of *vqmA_RIMD_* to insert into pBR322-pBAD; *vqmA_RIMD_-3XFLAG* gBlock |
| pEVS-P*vqmA_RIMD_* 2 | CGGCGGGTACCGCTCGAGTTAATTAATCGAGCTCTAGGATGCATACGACCCTGAGCTCTCGGTATCTGATTGATACCTCTAACTTACTTAGCAGACCCTCGCTATGTAATTTAGAACCTCTTCTACACGTCGACAAGATTATATCTTGTGTTGCCAGCCCGTCTTCAGGCTGGCTTTTTTTTTGTATCTCAAAAATGAAAACAATGGCATAAGCTGATATTATATCGGCCATCTCACCGTATCCTTACGTCAATCACCACGCTGAGTACTCTTGATCCATTTTTCTCCGCTCCCAGTGCCATCTGGTGCTATTTACCGAAAGCATGATCAAGGAATAACACTGCGTAGATGACGAAAGAATCCATGAAGTGAGCATACTGACCTGCATAAAGCAGGCAATGTATCCAAGTCATGTCTGAATGCTGGTGTAAACACAGCTGATGGAGAGATGGCTTGGGGCCAAATAGCAGTAATGACTAAAAAAATTTCATTCATTATTAACGGCCAGG | P*vqmA_RIMD_* for insertion into pEVS-*lux* |
| ODgBlock12 | GATGGCTTGGGGCCAAATAGCAGTAGTGAGTCCTCTTGTGAATATTCCAGTCAATACCATAGAACAGTCGCTCCTCCGACAGCTACCGGGTTGTTGGGGGTGCAAAGATAAAGATTCGGTATTCCGTTATGTGAATCAGGAATACGCCGAATTATTGGGCCATGCCTCACCAGAAGAGTGCATTGGTAAAACTGACTTCGAGATGTCGAGCCCAACCACTGAATGCGCACAAGAATTTCAACGCCAAGATAAACATGTGATCGAAACGGGCGAATCGTTGAAGATCCTTGATATTCATCCTTACCCTGATGGGCGTTGGCGCGCACATATTTTCACGAAAACCCCTTGGCGAGATGAACAAGGCAACACCCTCGGCACCATTTTTTATGGCCGAGAGTTAACCGACACCGCCGTGATTGAAGTGGGCTACTGGGTGTGTCGAGCCATCGGTACAGACATGAACCATCAATCCATTTTCCGCTTTTCGAATCTGAATCCGAAACCAGAAAAGCTCACATGCCGCGAGCAAGAAACGCTATTTTTACTGCTTTACGGCAAAAAACCTCAGTTTATTTCTCAAGTGATGGGCATCTCCACAAAAACGGTAGAAGGGCACGTCGCACGTCTAAGAAACAAGTTTGAAGCGAATAGTAAGAACGAGCTGATCGATAAAGCAATGGAGGCAGGCTATGGATCTGTTGTACCCAAAACGCTGCTTAAACATCAGCTTTCTGTTGTTTTGAATGGAGAGCGCGACTACAAAGACCATGACGGTGATTATAAAGATCATGATATCGACTACAAAGATGACGATAAATAACGCCACGCCGCTTTATCTTTATAAACA | *vqmA_RIMD_-3XFLAG* insert to make *V. parahaemolyticus vqmA-3XFLAG* pRE112 plasmids and pBAD-*vqmA_RIMD_*-*3XFLAG* |
| ODgBlock18 | TTTTTTGTTGATTCAATATCGTCATGCTTATTTATCGTCATCTTTGTAGTCGATATCATGATCTTTATAATCACCGTCATGGTCTTTGTAGTCCACCTTCTGCCGCTCTTCTTTACCATTCCATGCTTGTAGTTTGCGGTAAATCGTTGACGGACTGACATCAAGATACCCAGCGGCTCTTGGAATATTGCCGTCACACGCCTCAATCGCTTGCTCAATCGCCATTTTCTCTGTCATCCATAGCGGCATGATCTCTGAAACAGTCATCGCTTTTGGCTCAATCAGTTTAGATACAGATGGCCTATCTAACGGTTGATTCAGCGGCGGTGGCAACATATCTAACGTAATTTCTTTGCCGTTGTTGAGCACCACGATATTGCGTAGTACGTTTTGTAGTTGTCGGACGTTACCCGGCCACTCATAACTGTTAAAGCGGTCAATCACTTCTTGAGAAAAGCGGACAAAGTTTTTGCCCTCTTCATGAGACATGTAGCCTAACAGTGAGTACGCAATTTCAATCACATCTTCACCACGTTCGCGAAGAGGCGGAAGATGCAAAGGAATCACGTATAAACGGTAGTATAAGTCTTCGCGGAAACGCCCTTCTTGAACTTCTTTCCAAGGGTCTCGGTTGGTCGCACAAACGAAGCGCACGTCCACACTCTTCATTTTTGAAGAGCCCACTTTCTGGAACGTACCGGTTTGGATGAATCGCAATAATTTGGTTTGTAAGTCCAAATCCATTTCACACAATTCATCGAGGAACAACGTGCCACCATCGGCTAACTCAGCGGCACCTTGACGGTCATTTGCCGCACCAGTAAATGCCCCTTTTACGTGACCAAATAGTTCACTTTCGATTAAGTCTTTAGGTATGGCCGCACAGTTGATGGCAATAAACGGCTTATCACCACGTTTGCTTGCAGCGTGGATGGCTTCTGCGCATACCTCTTTACCCGTACCACTTTCACCAGTAATAAAGATACTCGCTTTACTGCTAGCGGCAGAGTCAATGGTGCGATAGACCTGCTGCATGGTTTGGCTGCTGCCGATAAAACCTTGGTAATTCTGATTACCCGGATTATCTGCTTCATTTTTTAGCTTAGTTGCTTTGCGAATTGCGTTGTTGACCGTAACACGCAGACGGTCTGCTTCACACGGTTTGATCAAAAAGTCTTGAGAGCCATGACGCATCGCTTCTACCGCTGTATCGATAGAGCCATGAGCCGTCATGAAAATGATCGGCACATCTGGATGACTCTTTTTAACGGCGTGCAAAACATCCATCCCCGTCATATCTGGCAGACGAAGATCGAGAAGAATAAGATCTGGAATTCGATGATTGAGACTTTCAATGGCATCTCTACCTGTACCGACAATATTAATGTCGATCCCTAACGGCGTGAGGTACGAACGGTATAACGCCGCAACCGATGCTGTATCCTCAACCAT | *luxO_RIMD_-3XFLAG* for insertion into pRE112 |
| ODgBlock19 | TTTTTTGTTGATTCAATATCGTCATGCTTATTTATCGTCATCTTTGTAGTCGATATCATGATCTTTATAATCACCGTCATGGTCTTTGTAGTCCACCTTCTGCCGCTCTTCTTTACCATTCCATGCTTGTAGTTTGCGGTAAATCGTTGACGGACTGACATCAAGATACCCAGCGGCTCTTGGAATATTGCCGTCACACGCCTCAATCGCTTGCTCAATCGCCATTTTCTCTGTCATCCATAGCGGCATGATCTCTGAAACAGTCATCGCTTTTGGCTCAATCAGTTTAGATACAGATGGCCTATCTAACGGTTGATTCAGCGGCGGTGGCAACATATCTAACGTAATTTCTTTGCCGTTGTTGAGCACCACGATATTGCGTAGTACGTTTTGTAGTTGTCGGACGTTACCCGGCCACTCATAACTGTTAAAGCGGTCAATCACTTCTTGAGAAAAGCGGACAAAGTTTTTGCCCTCTTCATGAGACATGTAGCCTAACAGTGAGTACGCAATTTCAATCACATCTTCACCACGTTCGCGAAGAGGCGGAAGATGCAAAGGAATCACGTATAAACGGTAGTATAAGTCTTCGCGGAAACGCCCTTCTTGAACTTCTTTCCAAGGGTCTCGGTTGGTCGCACAAACGAAGCGCACGTCCACACTCTTCATTTTTGAAGAGCCCACTTTCTGGAACGTACCGGTTTGGATGAATCGCAATAATTTGGTTTGTAAGTCCAAATCCATTTCACACAATTCATCGAGGAACAACGTGCCACCATCGGCTAACTCAGCGGCACCTTGACGGTCATTTGCCGCACCAGTAAATGCCCCTTTTACGTGACCAAATAGTTCACTTTCGATTAAGTCTTTAGGTATGGCCGCACAGTTGATGGCAATAAACGGCTTATCACCACGTTTGCTTGCAGCGTGGATGGCTTCTGCGCATACCTCTTTACCCGTACCACTTTCACCAGTAATAAAGATACTCGCTTTACTGCTAGCGGCAGAGTCAATGGTGCGATAGACCTGCTGCATGGTTTGGCTGCTGCCGATAAAACCTTGGTAATTCTGATTACCCGGATTATCTGCTTCATTTTTTAGCTTAGTTGCTTTGCGAATTGCGTTGTTGACCGTAACACGCAGACGGTCTGCTTCACACGGTTTGATCAAAAAGTCTTGAGAGCCATGAGCCGTCATGAAAATGATCGGCACATCTGGATGACTCTTTTTAACGGCGTGCAAAACATCCATCCCCGTCATATCTGGCAGACGAAGATCGAGAAGAATAAGATCTGGAATTCGATGATTGAGACTTTCAATGGCATCTCTACCTGTACCGACAATATTAATGTCGATCCCTAACGGCGTGAGGTACGAACGGTATAACGCCGCAACCGATGCTGTATCCTCAACCAT | *luxO_882_-3XFLAG* for insertion into pRE112 |
