# Supplementary material for: Natural silencing of quorum-sensing activity protects Vibrio parahaemolyticus from lysis by an autoinducer-detecting phage
Source: PLoS Genet. 2023 Jul 31;19(7):e1010809. doi: 10.1371/journal.pgen.1010809 (PMC10426928; doi:10.1371/journal.pgen.1010809)
Supplement: S4 Table — (DOCX) [file pgen.1010809.s004.docx]

##### Table S4 Plasmids used in this study.

| **Plasmid name (informal)** | **Strain ID (formal)** | **Marker, Origin** | **Source** |
| --- | --- | --- | --- |
| VP882 Ctr::*cm* | Ec-OD603 | Cm, VP882 | This study, courtesy of G. Beggs |
| VP882 *qtip*::*cm* | Ec-OD587 | Cm, VP882 | This study |
| P*gp69-lux* | JSS-1220 | Kan, p15A | [8] |
| P*qrr*3*-mRuby3* | BB-Ec0914 | Cm, p15A | [32] |
| P*luxC-CDABE* | BB-Ec0224 | Tet, pLAFR | [14] |
| pBAD-*vqmA_Phage_* | JSS-852 | Kan, p15A | [8] |
| *HIS-HALO-cI_VP882_-*P*q-lux* | JSS-3410 | Amp, Kan, pBR322 | This study |
| P*vqmR_Vc_*-*lux* | Ec-OD119 | Kan, p15A | [8] |
| P*vqmR_RIMD_*-*lux* | Ec-OD545 | Kan, p15A | This study |
| P*vqmA_RIMD_*-*lux* | Ec-OD389 | Kan, p15A | This study |
| P*qtip-lux* | Ec-OD567 | Kan, p15A | This study |
| pBAD-*vqmA_RIMD_-3XFLAG* | Ec-OD545 | Amp, pBR322 | This study |
| pBAD-*vqmA_Vc_-3XFLAG* | BB-Ec0042 | Amp, pBR322 | [13] |
| pRE112-*vqmA_882_-3XFLAG* | Ec-OD498 | Cm, oriR6kγ | This study |
| pRE112-*vqmA_RIMD_-3XFLAG* | Ec-OD499 | Cm, oriR6kγ | This study |
| pRE112-P*vqmA_882_-vqmA_882_-3XFLAG* | Ec-OD500 | Cm, oriR6kγ | This study |
| pRE112-*vqmR^+^-* P*vqmA_882_-vqmA_882_-3XFLAG* | Ec-OD501 | Cm, oriR6kγ | This study |
| pRE112- P*vqmA_Vc_-vqmA_RIMD_-3XFLAG* | Ec-OD521 | Cm, oriR6kγ | This study |
| pRE112- *luxO_882_*::*luxO_RIMD_* | Ec-OD525 | Cm, oriR6kγ | This study |
| pRE112-*luxO^D61E^* | FJS-S113 | Cm, oriR6kγ | This study, courtesy of F. Santoriello |
| pRE112-*luxO^D61A^* | FJS-S114 | Cm, oriR6kγ | This study, courtesy of F. Santoriello |
| pRE112-*luxO_882_^D61E^* | Ec-OD599 | Cm, oriR6kγ | This study |
| pRE112-*luxO_882_^D61A^* | EcOD-600 | Cm, oriR6kγ | This study |
| pRE112- *luxO_882_*::*luxO_RIMD_*-*3XFLAG* | EcOD-601 | Cm, oriR6kγ | This study |
| pRE112- *luxO_882_*::*luxO_882_*-*3XFLAG* | EcOD-602 | Cm, oriR6kγ | This study |
| pKAS- P*vqmA_RIMD_-vqmA_Vc_-3XFLAG* | EcOD-550 | Amp, oriR6kγ | This study |
