# Supplementary material for: Natural silencing of quorum-sensing activity protects Vibrio parahaemolyticus from lysis by an autoinducer-detecting phage
Source: PLoS Genet. 2023 Jul 31;19(7):e1010809. doi: 10.1371/journal.pgen.1010809 (PMC10426928; doi:10.1371/journal.pgen.1010809)
Supplement: S3 Data — (PPTX) [file pgen.1010809.s007.pptx]

## Slide 1
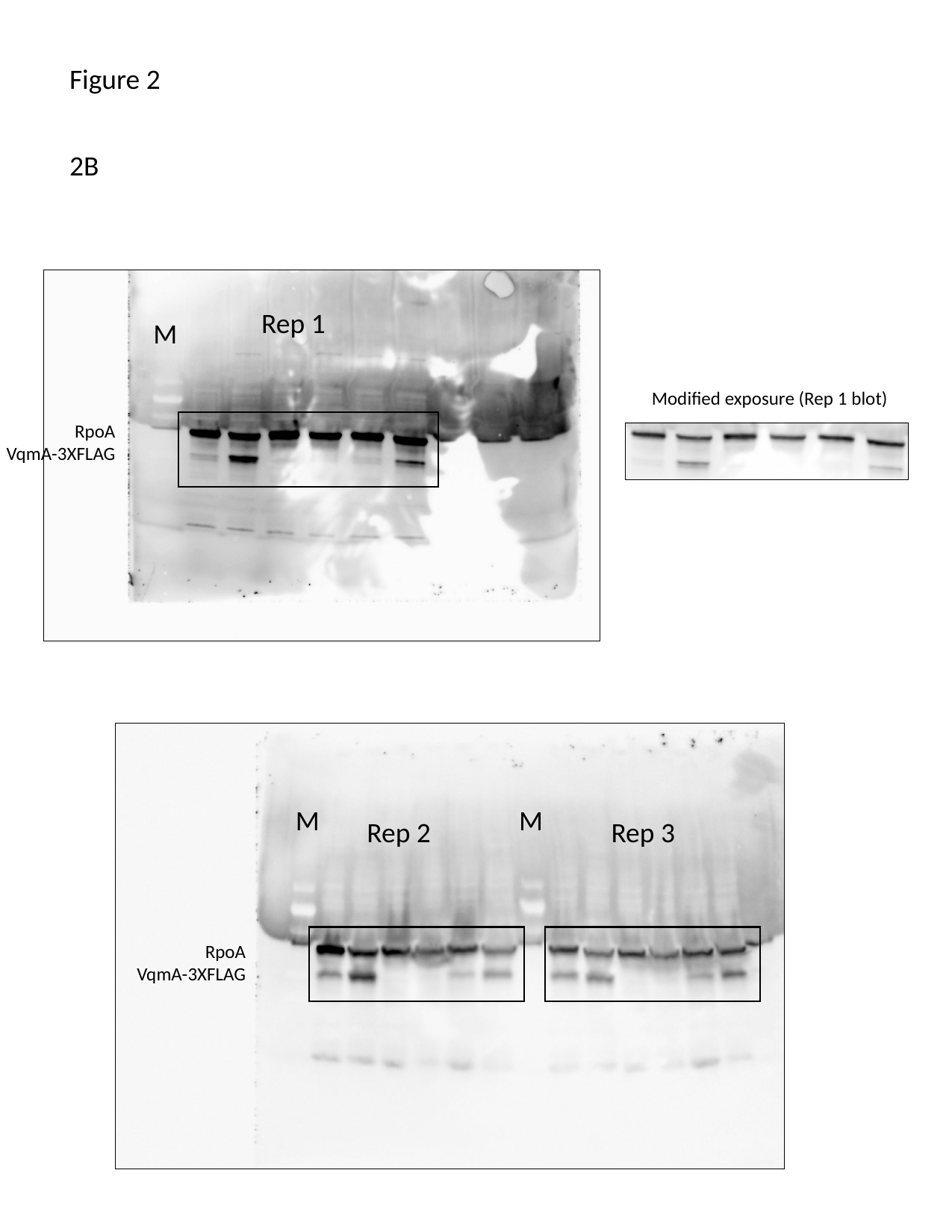

Figure 2
2B
Rep 1
M
Modified exposure (Rep 1 blot)
RpoA
VqmA-3XFLAG
M
M
Rep 2
Rep 3
RpoA
VqmA-3XFLAG

## Slide 2
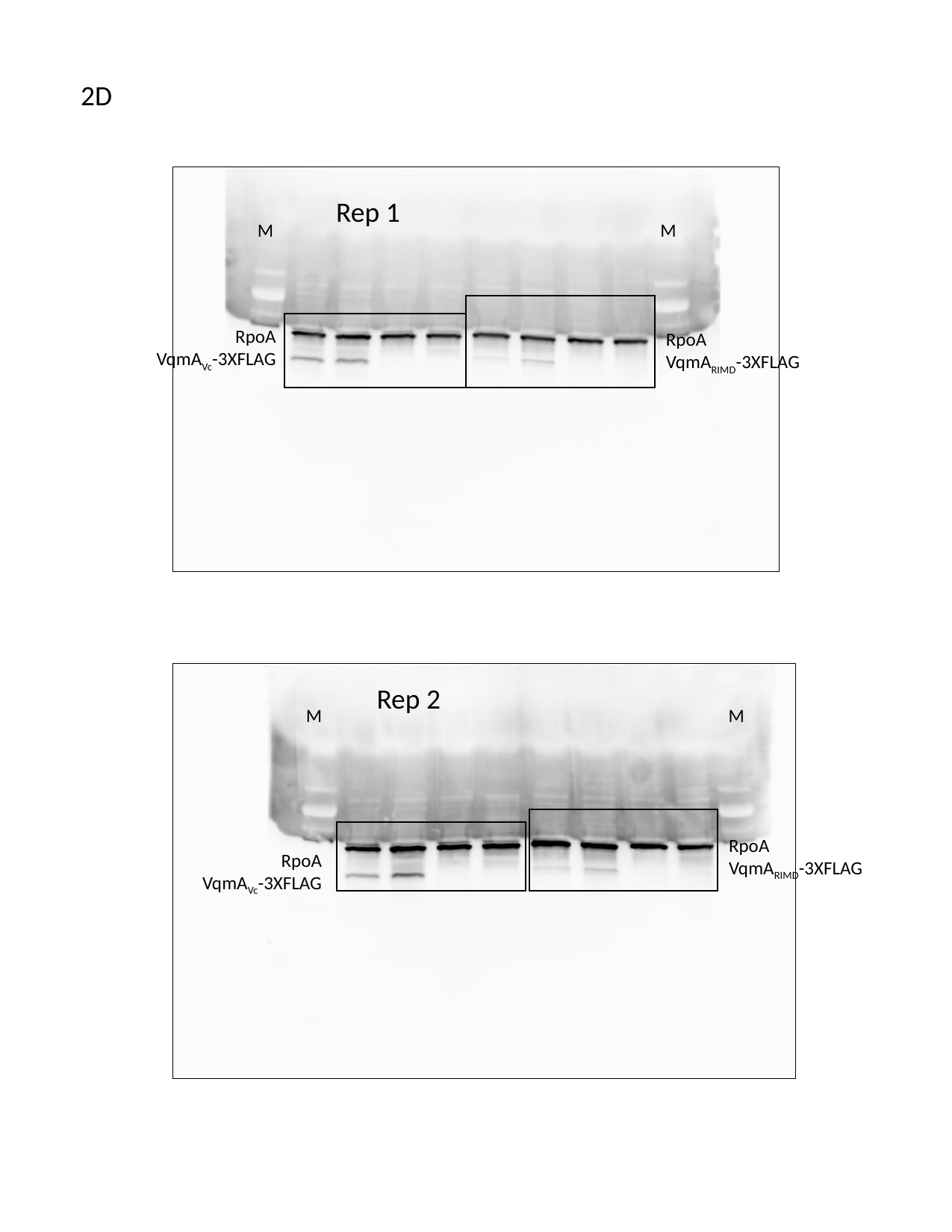

2D
Rep 1
M
M
RpoA
VqmAVc-3XFLAG
RpoA
VqmARIMD-3XFLAG
Rep 2
M
M
RpoA
VqmARIMD-3XFLAG
RpoA
VqmAVc-3XFLAG

## Slide 3
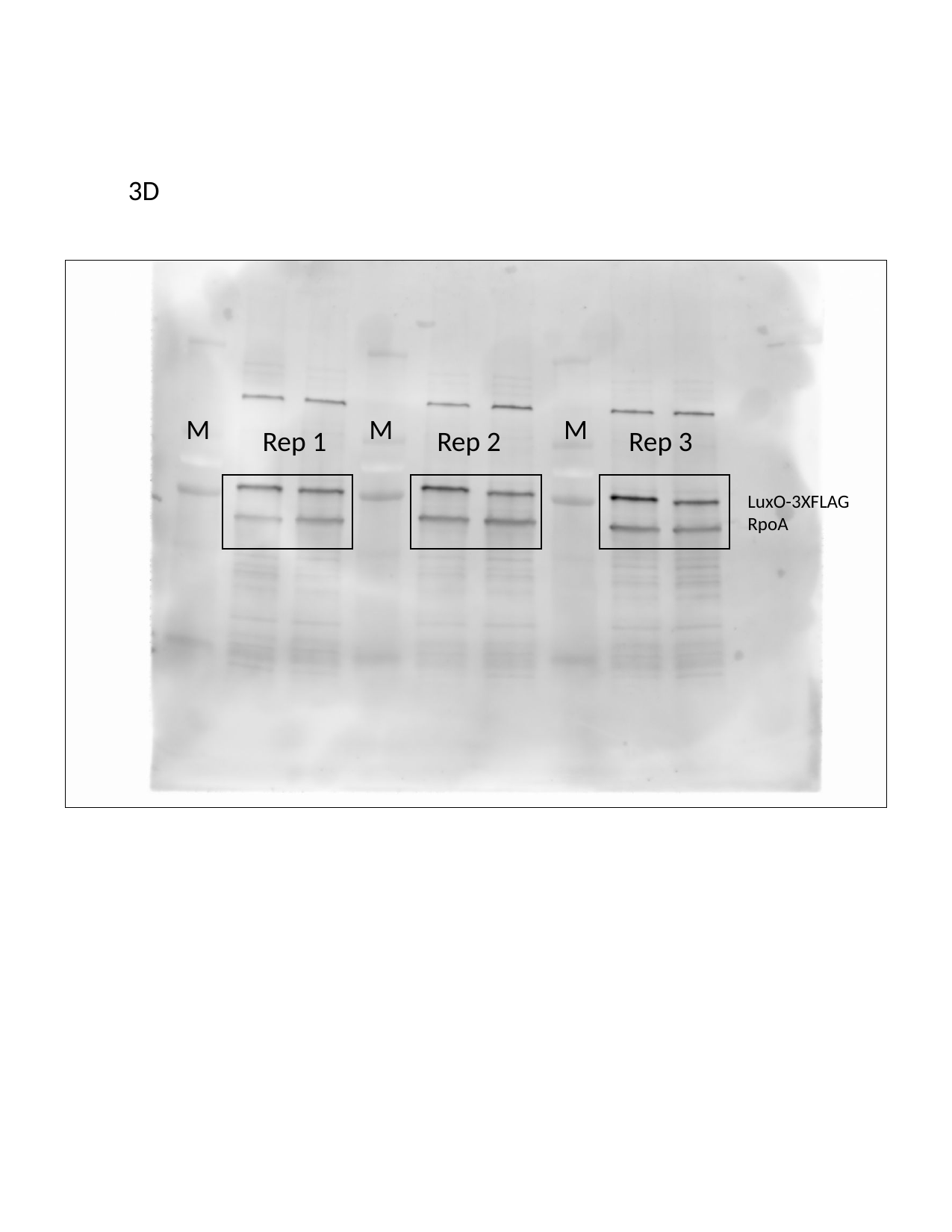

3D
M
M
M
Rep 1
Rep 2
Rep 3
LuxO-3XFLAG
RpoA

## Slide 4
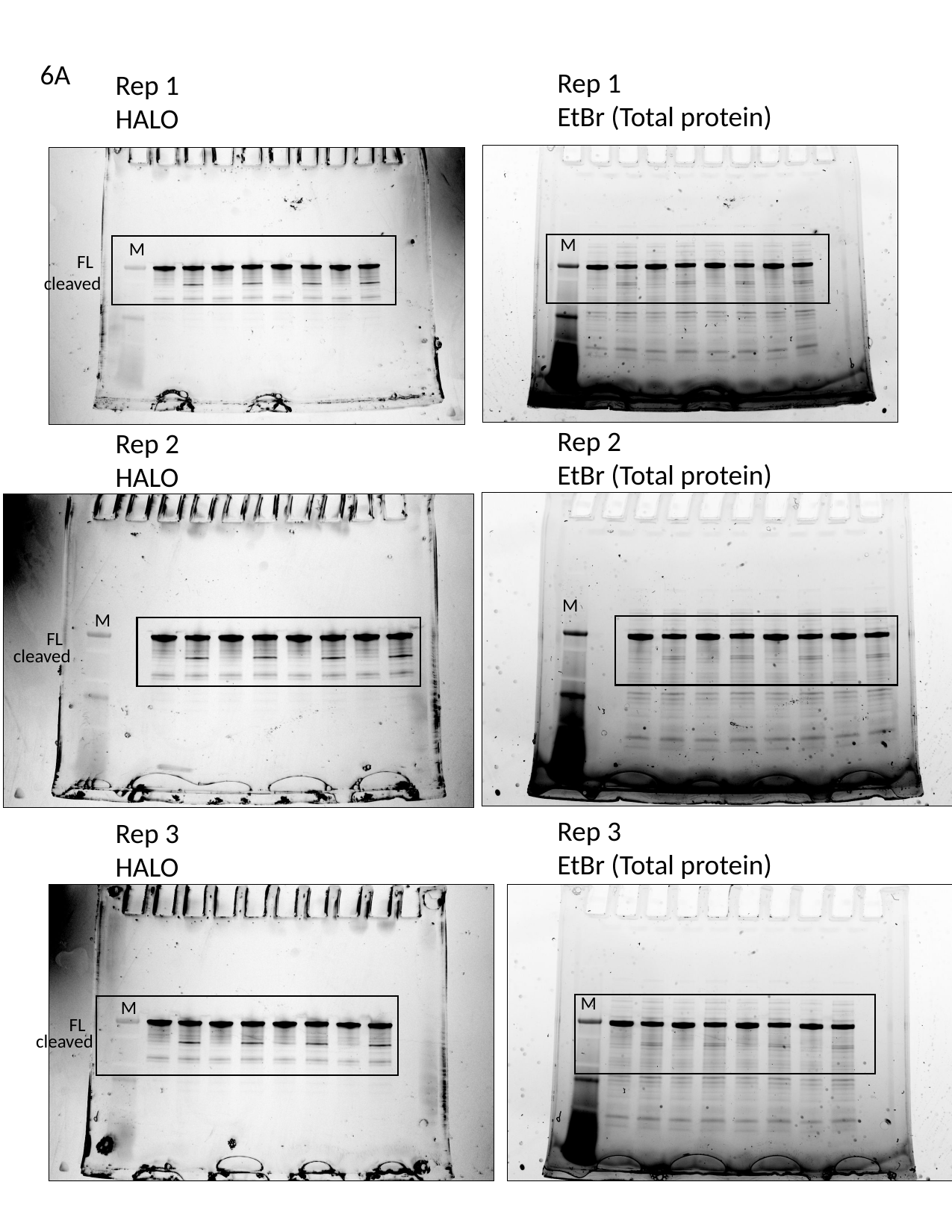

6A
Rep 1
EtBr (Total protein)
Rep 1 HALO
M
M
FL
cleaved
Rep 2
EtBr (Total protein)
Rep 2 HALO
M
M
FL
cleaved
Rep 3
EtBr (Total protein)
Rep 3 HALO
M
M
FL
cleaved

## Slide 5
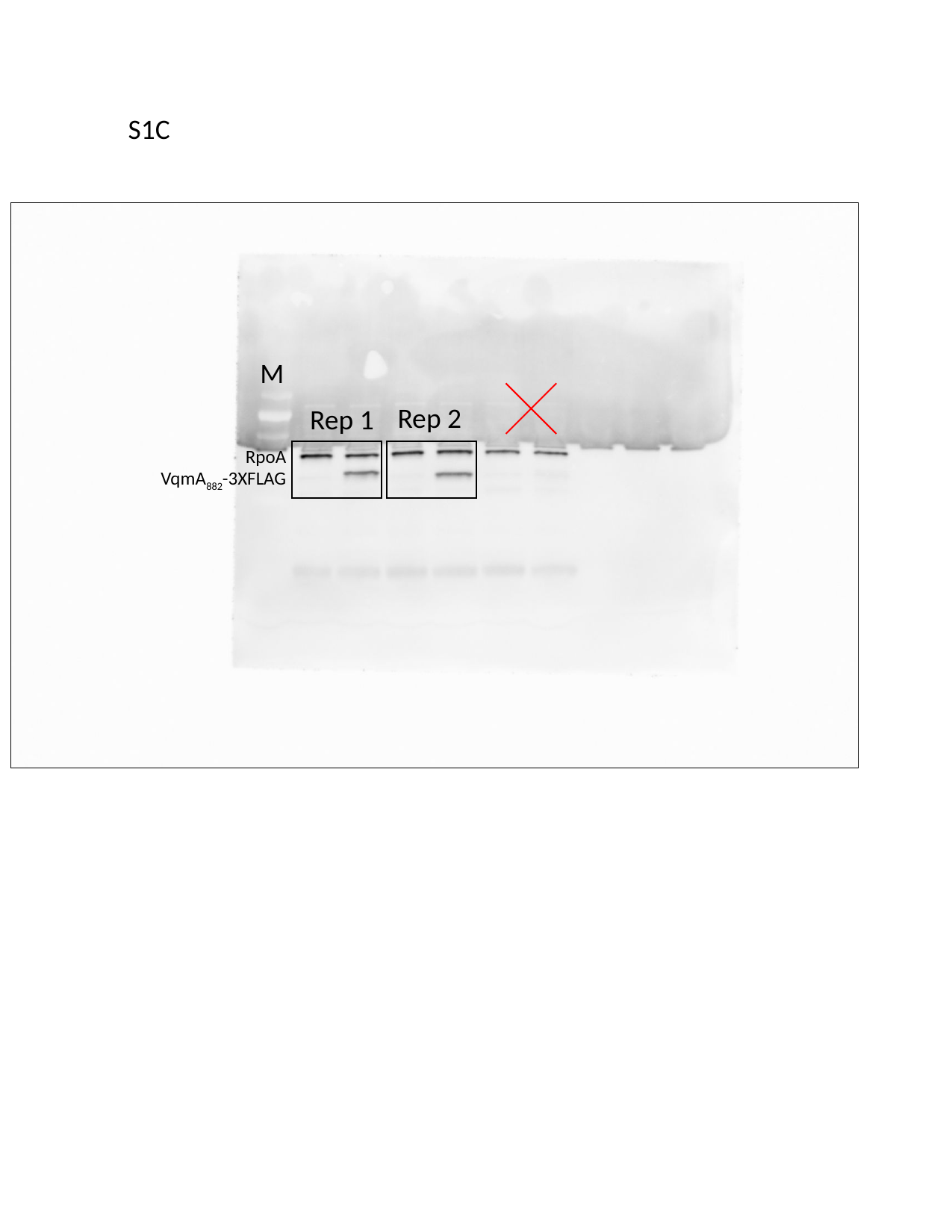

S1C
M
Rep 2
Rep 1
RpoA
VqmA882-3XFLAG
