# Supplementary figures and images for: Natural silencing of quorum-sensing activity protects Vibrio parahaemolyticus from lysis by an autoinducer-detecting phage
Source: PLoS Genet. 2023 Jul 31;19(7):e1010809. doi: 10.1371/journal.pgen.1010809 (PMC10426928; doi:10.1371/journal.pgen.1010809)

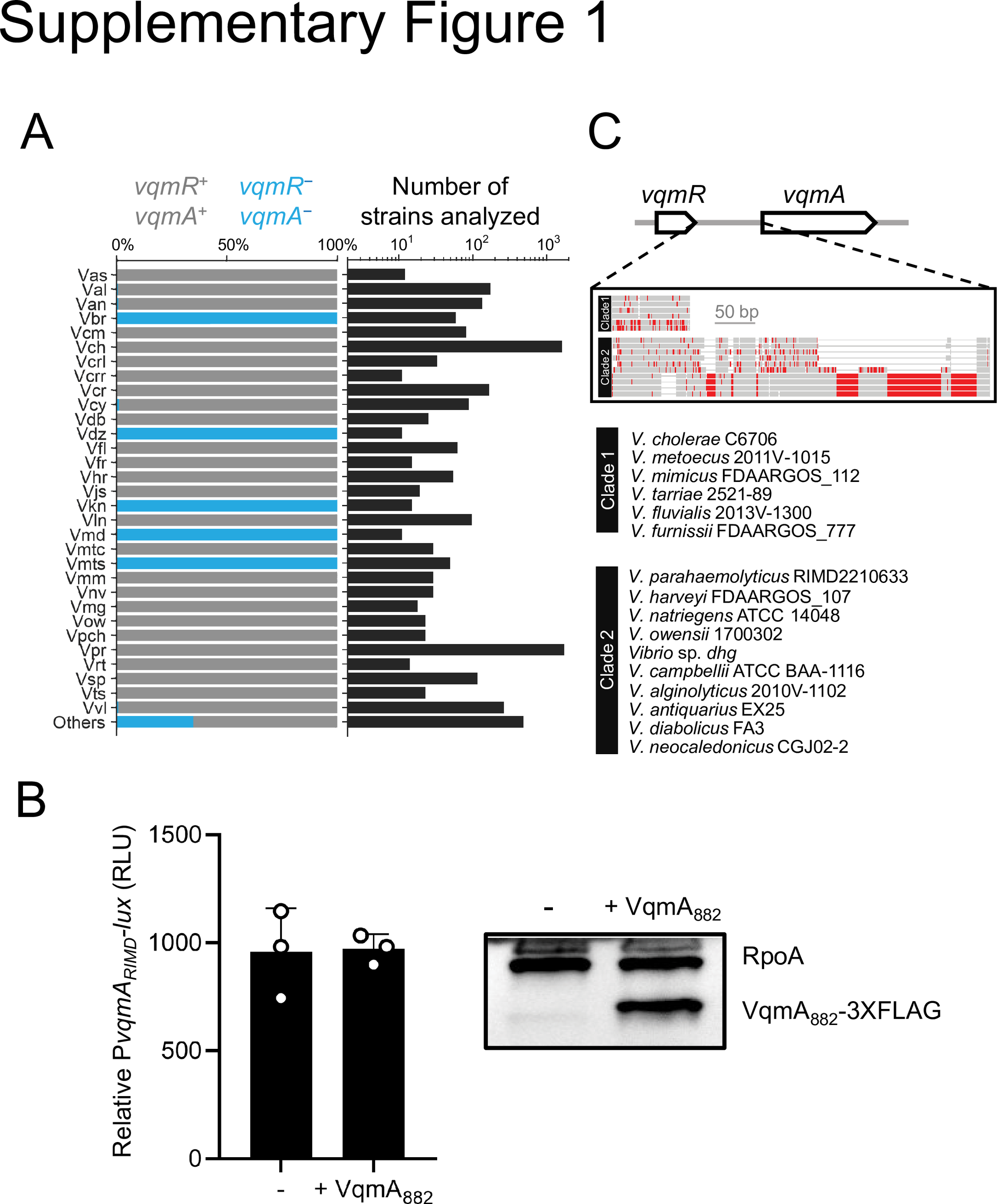

Supplement: S1 Fig — (A) Percentage of strains possessing (gray) or lacking (turquoise) vqmR-vqmA pairs (left), and number of strains analyzed (right) for the designated species. Vas: Vibrio aestuarianus. Val: Vibrio alginolyticus. Van: Vibrio anguillarum. Vbr: Vibrio breoganii. Vcm: Vibrio campbellii. Vch: Vibrio cholerae. Vcrl: Vibrio coralliilyticus. Vcrr: Vibrio coralliirubri. Vcr: Vibrio crassostreae. Vcy: Vibrio cyclitrophicus. Vdb: Vibrio diabolicus. Vdz: Vibrio diazotrophicus. Vfl: Vibrio fluvialis. Vfr: Vibrio furnissii. Vhr: Vibrio harveyi. Vjs: Vibrio jasicida. Vkn: Vibrio kanaloae. Vln: Vibrio lentus. Vmd: Vibrio mediterranei. Vmtc: Vibrio metoecus. Vmts: Vibrio metschnikovii. Vmm: Vibrio mimicus. Vnv: Vibrio navarrensis. Vmg: Vibrio nigripulchritudo. Vow: Vibrio owensii. Vpch: Vibrio paracholerae. Vpr: Vibrio parahaemolyticus. Vrt: Vibrio rotiferianus. Vsp: Vibrio splendidus. Vts: Vibrio tasmaniensis. Vvl: Vibrio vulnificus. (B) Left: Relative PvqmARIMD-lux output from E. coli carrying arabinose-inducible vqmA882-3XFLAG. The treatments - and + VqmA882 refer to water and 0.2% arabinose, respectively. RLU as in Fig 2B. Right: representative western blot of VqmA882-3XFLAG produced by the E. coli in the left panel. RpoA was used as the loading control. (C) Multiple DNA sequence alignment of the intergenic regions between vqmR and vqmA for the V. cholerae clade (Clade 1) and the V. parahaemolyticus clade (Clade 2). A representative strain (as designated) was chosen for each species in each clade. Thick gray or red bars indicate, respectively, nucleotides that are identical with or different from the consensus (>50% agreement among aligned sequences). Thin gray lines indicate gaps in the sequence alignments. Scale bar indicates 50 bp. Data in B are represented as means ± std with n = 3 biological replicates (left) and representative of two independent experiments (right). (TIF) [file pgen.1010809.s008.tif]

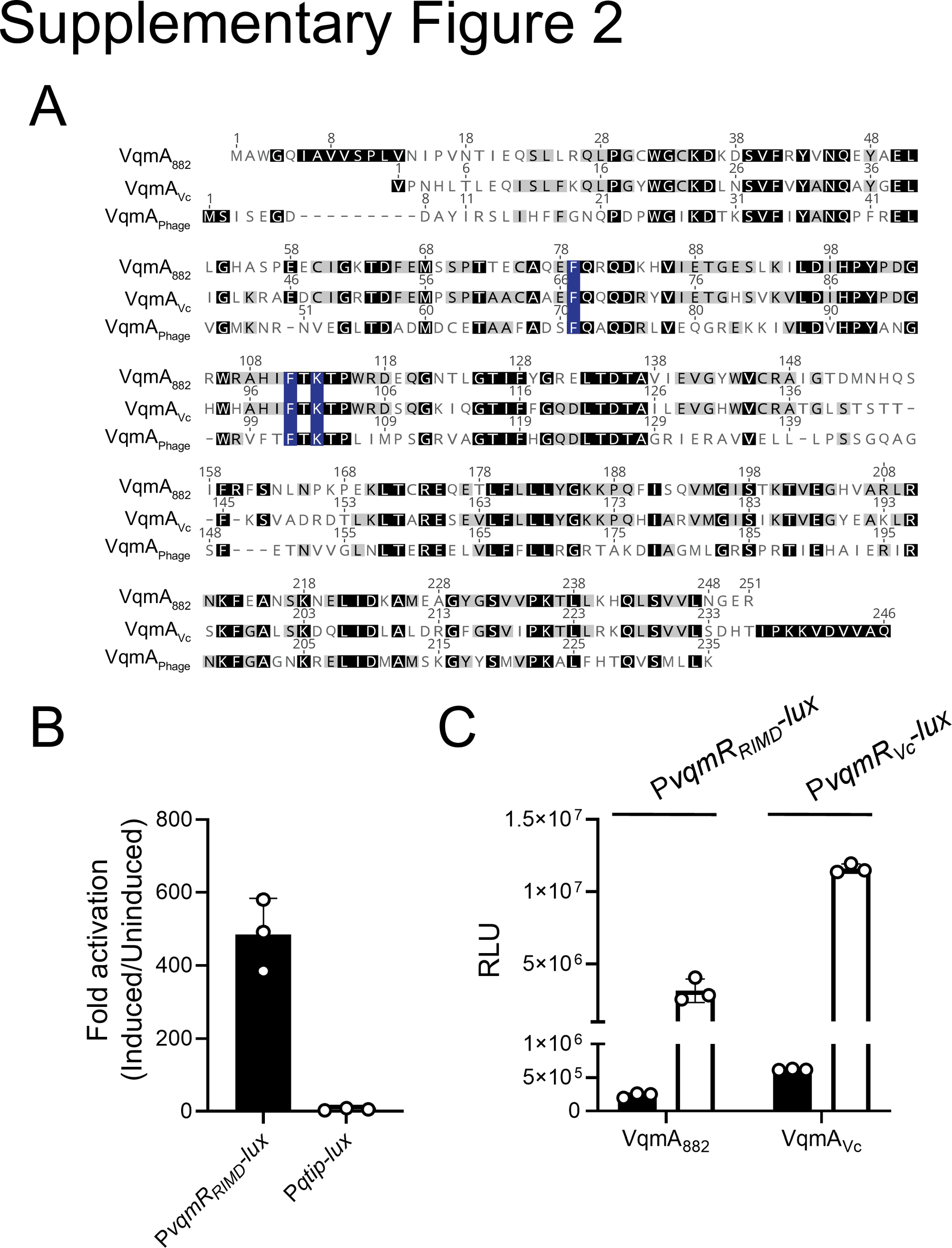

Supplement: S2 Fig — (A) Protein sequence alignment (ClustalW) showing V. parahaemolyticus strain 882 VqmA (VqmA882), V. cholerae VqmA (VqmAVc), and phage VP882 VqmA (VqmAPhage) proteins. Black and gray boxes designate identical and conserved residues, respectively. Numbering indicates amino acid positions. Blue boxes indicate key conserved DPO-binding residues from VqmAVc (F67, F99, and K101). (B) Relative fold activation of PvqmRRIMD-lux or Pqtip-lux from Δtdh E. coli harboring arabinose-inducible vqmA882-3XFLAG. Fold activation was calculated by dividing the RLU of induced cells (0.02% arabinose and 10 μM DPO) by the RLU of uninduced cells. (C) Relative PvqmRRIMD-lux and PvqmRVc-lux from Δtdh E. coli harboring arabinose-inducible vqmA882-3XFLAG (designated VqmA882) or vqmAVc-3XFLAG (designated VqmAVc), respectively. E. coli were treated with either water (black bars) or 10 μM DPO (white bars). All cells were treated with 0.02% arabinose. Data are represented as means ± std with n = 3 biological replicates (B, C). RLU as in Fig 2B (B, C). (TIF) [file pgen.1010809.s009.tif]

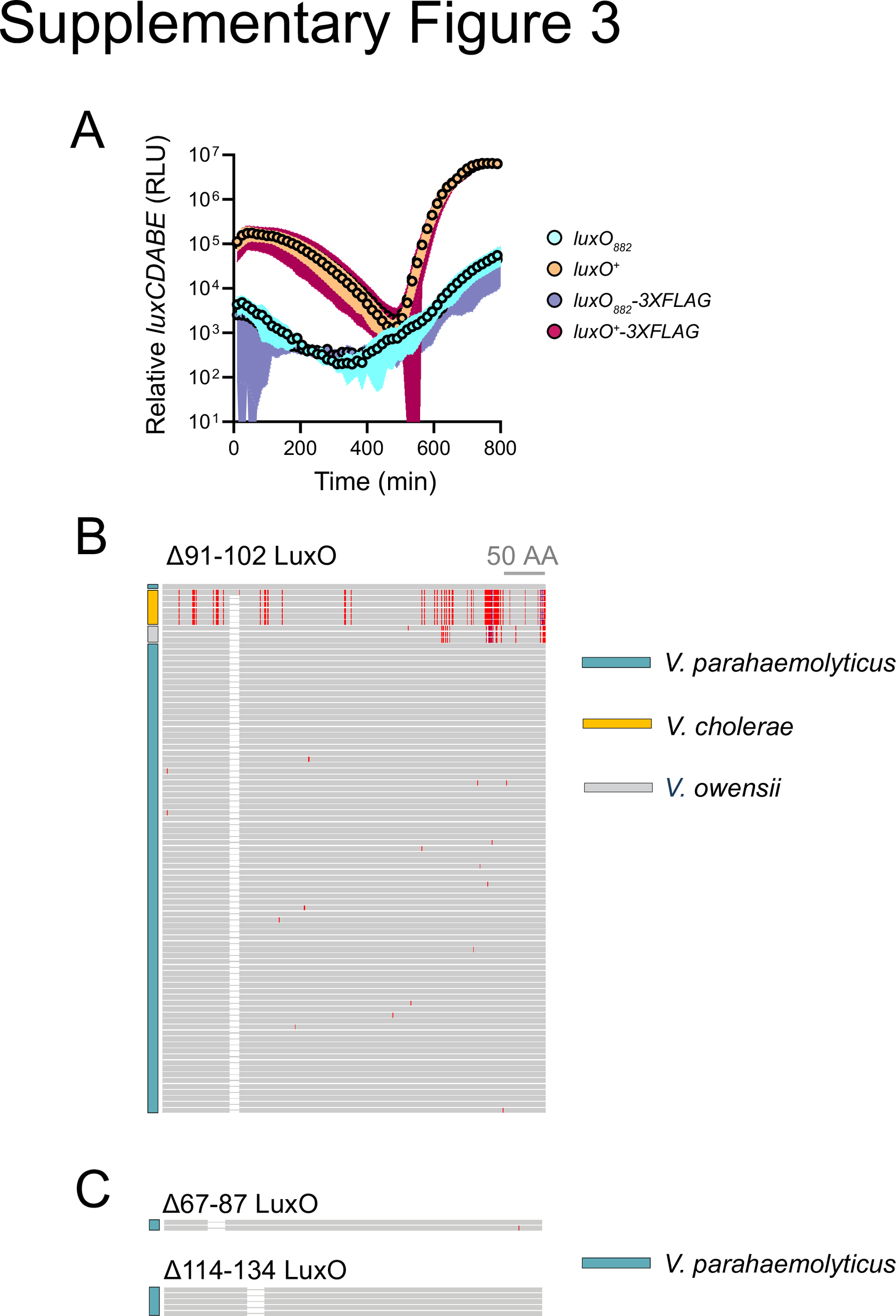

Supplement: S3 Fig — (A) Relative luxCDABE output over time from the 882 luxO882 (cyan), 882 luxO+ (orange), 882 luxO882-3XFLAG (purple), and 882 luxO+-3XFLAG (pink) strains. Data are represented as means ± std with n = 3 biological replicates. (B) Multiple amino acid sequence alignment of LuxO in Vibrio strains that carry the Δ91–102 luxO mutation. Gray and red vertical bars indicate, respectively, amino acids that are identical to or different from the consensus (>50% agreement among aligned sequences). White boxes indicate the 91–102 amino acid deletion. Blue vertical lines indicate insertions. Teal indicates V. parahaemolyticus strains, green indicates V. cholerae strains, and dark blue indicates V. owensii strains. Scale bar indicates 50 amino acids (abbreviated AA). All sequences are aligned with respect to the LuxO sequences of V. parahaemolyticus RIMD2210633 and V. cholerae C6706, which are shown in the first and second row, respectively. (C) As in (B), except the strains carry the Δ67–87 (top) or Δ114–134 (bottom) LuxO mutation. (TIF) [file pgen.1010809.s010.tif]

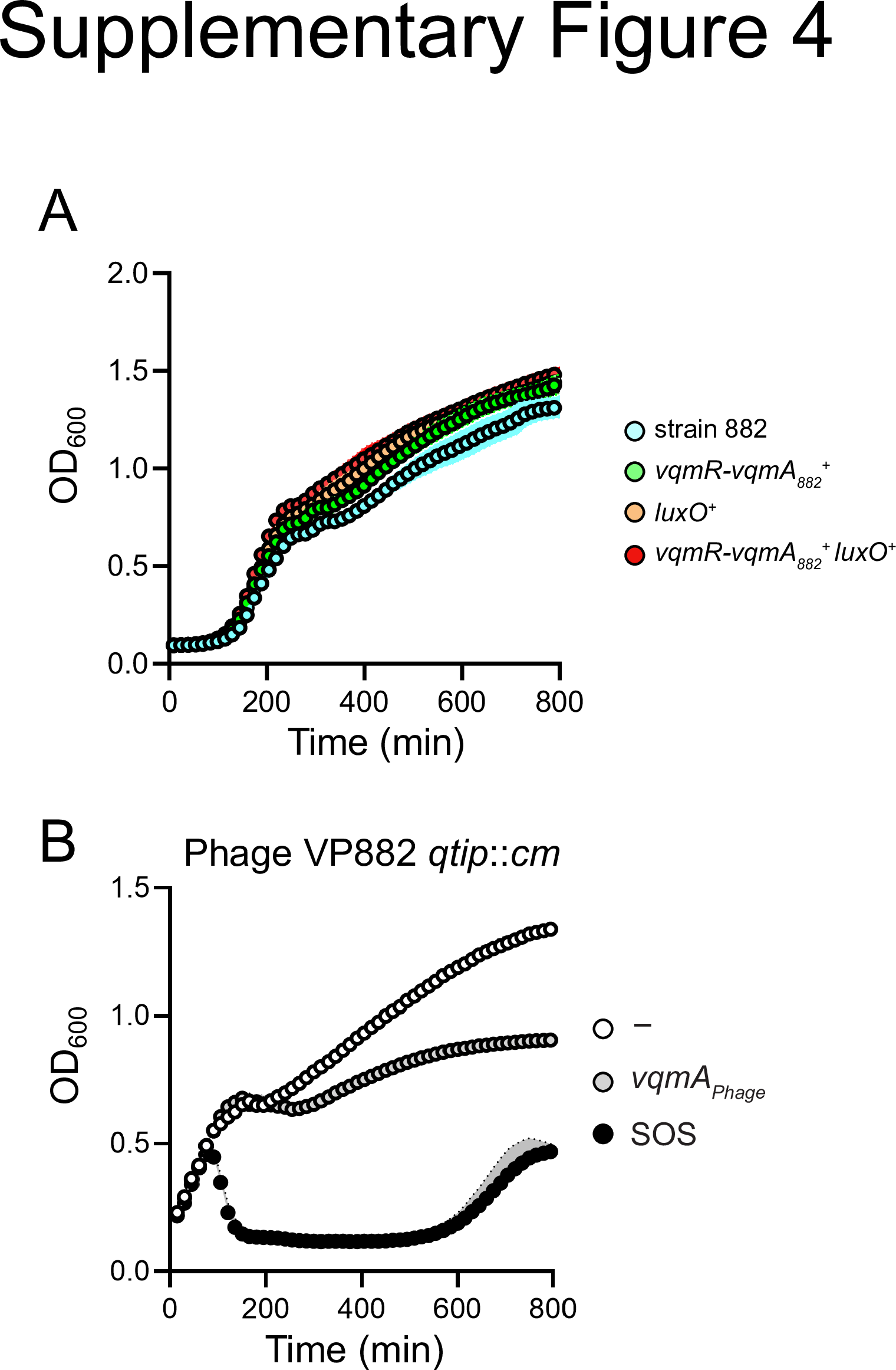

Supplement: S4 Fig — (A) Growth of the 882 parent (cyan), 882 vqmR-vqmA882+ (green), 882 luxO+ (orange), and 882 vqmR-vqmA882+ luxO+ (red) strains. (B) Growth of strain 882 harboring phage VP882 qtip::cm and arabinose-inducible vqmAPhage in medium treated with water (white), arabinose (gray), or ciprofloxacin (black). Arabinose (0.2%) was used to induce vqmAPhage expression, and ciprofloxacin (500 ng mL-1) was used to induce host SOS. Data are represented as means ± std with n = 3 biological replicates (A, B). (TIF) [file pgen.1010809.s011.tif]
