# Supplementary material for: Natural silencing of quorum-sensing activity protects Vibrio parahaemolyticus from lysis by an autoinducer-detecting phage
Source: PLoS Genet. 2023 Jul 31;19(7):e1010809. doi: 10.1371/journal.pgen.1010809 (PMC10426928; doi:10.1371/journal.pgen.1010809)
Supplement: Attachment — Submitted filename: Bassler_Response_to_reviewer.docx [file pgen.1010809.s012.docx]

Dear Dr Bassler,

Thank you very much for submitting your Research Article entitled 'Natural Silencing of Quorum-Sensing Activity Protects Vibrio parahaemolyticus from Lysis by an Autoinducer-Detecting Phage' to PLOS Genetics.

The manuscript was fully evaluated at the editorial level and by independent peer reviewers. All three reviewers appreciated the attention to an important topic but suggested some minor suggestions that we ask you address in a revised manuscript.

[…]

Reviewer's Responses to Questions

**Comments to the Authors: Please note here if the review is uploaded as an attachment.**

Reviewer #1: In recent years, there have been exciting new studies (particularly from the Bassler lab) focused on connections between quorum-sensing and symbioses of phage and their prokaryotic hosts. The Bassler lab recently reported a vibriophage VP882, capable of sensing host-produced QS signals that trigger prophage induction. Presumably host QS molecules act as a signal of high prey availability to a prophage. Here, Duddy et al. sequence the genome of the natural host of the VP882 prophage, and find that two host QS systems are disabled. They demonstrate that, if functionality of these systems is restored, virion production and host cell lysis by the phage are enhanced, suggesting that inactivation of host QS confers a fitness benefit to VP882 lysogens.

The data in this paper are beyond reproach and strongly support the conclusions. The paper is also very well-written and was a pleasure to read. I do not have any experimental requests, but have a few points of interest that could be clarified via additional discussion:

Thank you for the kind words and the constructive review.

1. Is there any correlation between Vibrio isolates that have VqmR-VqmA deletions and those that contain a resident prophage encoding a VqmA homolog?

No other Vibrios in current sequencing databases harbor a phage that encodes a VqmA homolog. We routinely search the databases for that possibility. We hesitate to speculate too strongly on any correlation, given the limitations in available sequencing data.

2. It seems that strain 882 produces DPO at high cell density, as restoration of VqmA-R results in cell density-dependent phenotypes. Is the DPO synthase known? It might be worth pointing out whether it’s present in strain 882.

Tdh (threonine dehydrogenase) is the DPO synthase. We have reported that previously as well as a potential biosynthetic route to DPO from threonine. Tdh is present in strain 882. We have now included this point in the main text and added the citation for the discovery and characterization of Tdh as the DPO synthase.

3. Could the mysterious signal activating transcription of VqmA phage be a direct target of Qrr-mediated translational silencing? Or conversely, is OpaR required for the observed QS-dependent phage phenotype?

It is certainly possible. As you can imagine, we are working maniacally to identify the pathway components that regulate *vqmA_Phage_* expression. Our strategy relies on a combination of Tn5 mutagenesis of the phage and the host and overexpression libraries, again of phage and host genes. We tested the Qrr sRNAs and OpaR in a reporter assay for direct regulation and, preliminarily, neither showed activity.

4. Once VP882 is established in a host, triggering prophage induction in response to high cell density might be disadvantageous if the producers are lysogenic kin, as these cells would presumably not constitute susceptible prey (since lysogens are typically resistant to superinfection by the phage they already harbor). Therefore it might benefit both the host and phage if the host QS systems are disabled, but phage VqmA remains competent to respond to QS signals from neighboring un-lysogenized cells.

Thank you for the insightful comment. The first point is similar to remarks made by Referee’s #2 and #3. We agree with you. We have added text to the revised discussion to make it more balanced. Previously, we suggested the host benefits by suppressing the phage lytic cascade. We now also include that an alternative is that the phage could benefit by suppressing lysis when no susceptible prey are nearby due to the host population being lysogenized and therefore immune to superinfection.

We do not currently know of a mechanism by which phage VP882 can discern already-infected from susceptible hosts since, at least with the Vibrio strains we have worked with so far, both types of hosts make equivalent amounts of the DPO autoinducer and so the phage would be “tuned in” to both lysogenized and non-lysogenized hosts.

5. Very minor, but it might be worth emphasizing multiple times that prophage VqmA is silent until the unknown signal is produced. I found myself wondering why phage VqmA wasn’t responding to HCD, and this took a while to realize.

Thank you.

In the revised version, this point now appears multiple times.

Reviewer #2: This is a well conducted and written study characterising loss of function mutations in Vibrio parahaemolyticus that prevent lysis by prophage that detect Quorum Sensing signals. The study is of general interest for understanding how phages regulate their behaviour in response to host ecology, and of more specific interest because the focus is on the natural host of the archetypal “QS eavesdropping” phage that has been extensively studied.I only have minor comments.

1. End of introduction. Phage could also be beneficial (acting as biowarfare agents), so suggest refrain from use of parasite.

Indeed. Also as suggested by Referees #1 and #3. We have added text to the revised discussion to make it more balanced. Previously, we suggested the host benefits by suppressing the phage lytic cascade. We now also include that an alternative is that the phage could benefit by suppressing lysis when no susceptible prey are nearby due to the host population being lysogenized and therefore immune to superinfection.

2. Any information linking mutations in vqmA, vqmQ or lux genes and prophage incidence in general across the approx. 5000 genomes?

We have performed a preliminary analysis of select bacterial genomes to explore whether there is a correlation between bacteria harboring defects in QS genes and harboring prophages. Specifically, we used PHASTER (https://phaster.ca/) to search for prophage sequences in bacterial genomes of two groups of *V. parahaemolyticus*: (1) 88 strains harboring *luxO* ^Δ91-102^, and (2) 88 other randomly selected strains harboring intact *luxO*. Our results showed that the number of prophage regions is not significantly different between the two groups (p-value=0.2; chi-square test). Further study is required to examine all ~5000 genomes to investigate a possible correlation between prophage presence and other QS defects.

It is worth noting that VP882 is the only phage in the 5,000 genomes that harbors the *vqmA* homolog. In other work, we have also discovered and reported phages in different bacteria with *luxR* QS genes and we are studying them now. We have not yet identified any other QS genes on phages. More generally, we have not identified any correlation among the phages that possess either *vqmA* or *luxR* QS genes. However, that could be a consequence of limited high-quality phage sequences available. We routinely search the databases for phages with QS genes in the hopes that we find more, and more interestingly, some correlation.

3. Interesting to carry outs test of associations between presence of defective Lux and VqmA systems across the genomes

We have performed a rudimentary analysis as suggested by the referee. We examined the coincidence of deletions or null mutations (denoted by superscript “–”) in three QS genes, *vqmA, luxO*, and *opaR/hapR*, across Vibrios. Our results are summarized in the Venn diagram below. We note that there is possibly a weak enrichment of *luxO*^–^ and/or *hapR*^–^ in the *vqmA*^–^ *Vibrio* strains compared to *vqmA*^+^ strains. We also note that we cannot tell if polymorphisms render QS proteins non-functional, so our analysis provides no information on that possibility. We provide this analysis for the referee’s interest but have chosen not to include it in the revised manuscript as we are not confident about weak “possible” correlations. Furthermore, an interpretation based on only these QS components is incomplete without considering other key QS regulators that govern their activity (e.g., CqsA-S; LuxS-PQ; LuxM-N; LuxU, Qrr sRNAs, Tdh, etc.), none of which were a focus of the present work.

4756

*vqmA^–^*

*luxO^–^*

*opaR/hapR^–^*

9

35

8

79

186

226

161

4. Page 13, first paragraph. Explain more clearly interactions between Lux and VqmA on phage gene expression. They cant be both additive and epistatic at the same time.

Correct. We thank the referee for pointing this out. It was confusing. We have removed the word epistasis and have clarified the point.

5. The implication throughout is that preventing lysis confers a fitness advantage to the host. But you don’t show this: bacterial growth isn’t affected by QS-induced lysis. Please clarify this pint in the discussion, linking back to point 1.

Regarding the fitness advantage (and see our response to this Referee’s point 1): We agree. We have revised the point and now note that phages can be parasites and they can also endow their hosts with beneficial traits.

Regarding cell growth: In the initial submission, we explicitly explained that our results show that phage lytic genes are upregulated and viral replication/phage production increases in this strain. However, at the population level, under our conditions, those changes are insufficient to detectably alter cell growth.

Reviewer #3: Duddy et al examine the sequence and signalling characteristics of a strain of Vibrio parahaemolyticus of interest because it is the source of a bacteriophage, VP882, encoding VqmAPhage, a protein homolog of the receptor for DPO, a quorum sensing (QS) molecule for Vibrios. In previous work, the Bassler lab first demonstrated that DPO activates a host-encoded protein, VqmA, which leads to group behaviors, including biofilm formation. In work on the phage homolog, they found that VqmAPhage could also bind DPO, and expression of this protein initiated a signalling cascade leading to induction of the phage lysogen. Here, they find that the normal VqmA host signalling is defective in the natural host of VP882, and explore the consequences of this. By “repairing” the mutations, they show that the variants found in this host help to keep the expression of phage genes low, presumably avoiding inappropriate induction of phage lytic growth. Comparative genomics on the components highlighted here provide some novel insights into evolution of the QS pathways among Vibrio strains, although the implications of these have not been fully explored.

1. P. 2, introduction: A rationale provided here for the induction of the VqmAphage pathway and phage lysis at high cell density is that this is a situation where “the probability of infecting the next host cell is maximized.” That would not work well if the other host cells in the vicinity are also lysogens. Given that it is not known what the inducing signal for VqmAphage is, might there be a signal that transmits information about sensitive, non-lysogenic hosts? While this is not a focus of this paper, the work here does suggest that lysogenizing Vibrios that have fully competent QS systems may create problems.

Thank you for the insightful comment. This point is similar to remarks made by Referee’s #1 and #2

As we wrote above: We agree with you. We have added text to the revised discussion to make it more balanced. Previously, we suggested the host benefits by suppressing the phage lytic cascade. We now also include that an alternative is that the phage could benefit by suppressing lysis when no susceptible prey are nearby due to the host population being lysogenized.

2. Presumably, previously published work on VqmAphage and its ability to activate synthesis of VqmR was not done in this host. It would be useful to spell that out someplace.

The referee is correct. In the revised manuscript, we now note that earlier work examining these interactions was not performed in this host strain.

3. In reporting the sequence changes in the 882 strain, the focus is on expressionf VqmA882. However, if VqmR is also absent, it would seem that signalling from VqmAphage to VqmR and thus to host behaviors would also be absent in this strain. In swaps into other Vibrios, just the intergenic region was traded, but it was unclear in the text exactly what was “repaired” in some of the experiments. For instance, in Fig. 2b, is it only the vqmA and upstream region that is restored? Is this then different from the strains used later in the paper (Fig. 4).

The referee’s understanding about expression is correct. Our results show that the gene, when expressed in a heterologous *E. coli* host, activates expression of a synthetic P*vqmR* reporter. However, in *V. parahaemolyticus* strain 882, the lack of the *vqmA*_882_ promoter precludes *vqmA* expression. On page 5-6 of our initial submission, we noted the same two points the referee mentions here. Specifically, “Thus, the *vqmA_882_* gene, if expressed in strain 882, would function analogously to VqmA_Vc_ in *V. cholerae*. However, the VqmR-VqmA QS system in strain 882 is defective for two reasons – because it lacks VqmR and it does not express *vqmA_882_*.”

In Figure 2b, indeed, only the intergenic upstream region was restored (i.e., the phenotype is VqmA^+^/VqmR^-^). In contrast, in all instances of the text where VqmA/R is indicated as repaired, both *vqmA/R* are restored (i.e., VqmA^+^/VqmR^+^ phenotype). As the referee notes, the pathway would not function without *vqmR* also being present. This point is clarified in the revised manuscript.

4. Is the statement “Of the >5000 sequenced strains, strain 882 is the only sequenced Vibrio that lacks vqmR but retains vqmA” referring to the phage version of vqmA? Is it also the only phage VqmA? Maybe it would be worth returning vqmR but not vqmA to the 882 genome, to see the effect of the lysogen in that context.

We thank the Referee for pointing out that we were unclear. The statement refers to the host *vqmA*. *V. parahaemolyticus* strain 882 is the only sequenced Vibrio that lacks *vqmR* but retains the host-encoded *vqmA*. We have clarified this point in the text. And, yes, strain 882 is also the only Vibrio to possess VqmA_Phage_. We have also made this point explicit in the revised text.

5. Fig. 2C , 2D, and S1C: These two clades of vqmR/vqmA are interesting. In Clade I, VqmA is expressed constitutively, and a reasonable -10 region seems to be present, although the lack of function for a swap of this sequence into RIMD suggests either something further upstream or possibly another problem. If the transcription start site is known for that, I might be mentioned. In Clade II, no decent conserved -10 is obvious. Is there any evidence where transcription actually starts? Or that transcription (and not translation) is responding to cell density? If the swap to look at what is needed for expression includes the upstream vqmR gene, does this work better? What if it includes first part of the vqmA genes?

The Referee has asked us terrific questions that we are pursuing now. The present work revealed the differences in host VqmA production among the strains. We do not yet know the molecular mechanism responsible.

6. Fig. 3B, Fig. S3B, Fig. 5B, Fig. 6C: It is not very easy to see what colors are what in many of these graphs (luxO882 and luxOD61A are particularly difficult to tell apart in 3B, for instance). It was also not entirely clear that luxOD61E is full-length luxO, while luxO882D61E is the one with the internal deletion. Maybe spell out more in the legend.

As suggested, we changed the color scheme to make the differences more pronounced. We also clarified the differences between the alleles in the legend.

7. Fig. 3F: Since these are all strains with the deletion (other than RIMD2210633), it is difficult to really follow how many times the authors think this deletion arose. It might be more useful to look at trees and close relatives for a few other Vibrios not closely related. If this happened multiple times, aside from some expectation of selection for LCD state, does the DNA sequence around this deletion reveal a likely recombination hot-spot?

Indeed, this is an extremely interesting idea. Figure 3F emphasizes the point that the Δ91-102 deletion in *luxO* that is present in strain 882 is unlikely to have arisen from a single common ancestor. We have not yet investigated how many times the same or similar mutations occurred. Such an investigation would undoubtedly be important for understanding the evolution of QS states in bacteria, in general, however, it seems beyond the scope of the present work which we want to keep focused on host-phage interactions.

8. If LuxO is constitutively active in these strains, locked into an LCD state, is there any loss/change in upstream signalling machinery or downstream output (Qrr sRNAs or OpaR?)? This is an interesting observation but does suggest a very different lifestyle (or maybe a more active VqmA system?).

This comment too is a fascinating point about Vibrio evolution (and presumably how they thrive in their various niches). To our knowledge, no one has systematically investigated the upstream signaling capabilities of naturally occurring vibrio strains that are LCD locked. We think, now that we have strain 882, coupled with the fact that now, a few other naturally occurring LCD locked Vibrio strains exist in labs so we should be able to acquire them, mounting such an investigation would be very worthwhile.

9. Fig. 4 and epistasis of luxO+. A bit more discussion of why luxO+ is needed for the vqmR/A genes to have any effect would be useful. In Fig. 1A, the implication is that there are parallel but independent inputs into “group behaviors”. Here, the data focuses on phage genes; do group behavior genes show a similar pattern of luxO epistasis? Has that been looked at before in other Vibrios, or does the observation here suggest the evolution of different wiring, maybe due to the LuxO constitutive derivative?

We thank the Referee for noting that our text was confusing. Referee #2 (point 4) made a similar comment. We have removed the word epistasis and have clarified the point.

10. Fig. S3B: Again, hard to see color differences for V. cholerae and parahaemolyticus. Since only derivatives with this deletion are shown, other changes are not particularly informative (the species differ). Are there other differences relative to luxO+ strains?

As suggested, we have changed the colors to make the differences more pronounced. Performing whole genome comparisons between *luxO*^-^ and *luxO*^+^ strains would require whole genome annotations and alignments for all 5000+ genomes, which is computationally intensive and beyond the scope of the current study. Nevertheless, we compared select genes, namely *vqmA* and *hapR*, in *luxO*^-^ and *luxO*^+^ strains. We provide that analysis in answering an earlier question to Referee 2 (point 3), the relevant portion of which we have excerpted below:

Our results are summarized in the Venn diagram below. We note that there is possibly a weak enrichment of *luxO*^–^ and/or *hapR*^–^ in the *vqmA*^–^ *Vibrio* strains compared to *vqmA*^+^ strains. We also note that we cannot tell if polymorphisms render QS proteins non-functional, so our analysis provides no information on that possibility. We provide this analysis for the referee’s interest but have chosen not to include it in the revised manuscript as we are not confident about weak “possible” correlations. Furthermore, an interpretation based on only these QS components is incomplete without considering other key QS regulators that govern their activity (e.g., CqsA-S; LuxS-PQ; LuxM-N; LuxU, Qrr sRNAs, Tdh, etc.), none of which were a focus of the present work.

4756

*vqmA^–^*

*luxO^–^*

*opaR/hapR^–^*

9

35

8

79

186

226

161

11. Discussion: Is it really true that the same phage was found in Salmonella and Shewanella strains? How clear is it that this is real? Does the phage infect Salmonella? Shewanella? Is it possible these sequences were contaminants?

Yes, it sure seems so! The predicted phages were uncovered as contigs from BLASTn searches using phage VP882 as the query. Unfortunately, despite repeated attempts, we have been unable to obtain these isolates. Thus, they have not been experimentally confirmed as phages nor have their host ranges been explored. Based on the data available from the respective sequencing projects (<https://www.ncbi.nlm.nih.gov/nuccore/AAEKWQ010000030.1/> for *Salmonella enterica* strain PNUSAS009922 and <https://www.ncbi.nlm.nih.gov/nuccore/LTBI01000116.1/> for *Shewanella algae* strain CLS1), we presume they were both sequenced from isolates. We can confirm this in the case of *Salmonella*, which was sequenced in the US by CDC. We, therefore, believe it is unlikely they are contaminants, but, of course, we cannot fully exclude this possibility until we acquire the isolates and do the work ourselves.

**Have all data underlying the figures and results presented in the manuscript been provided?**
Large-scale datasets should be made available via a public repository as described in the *PLOS Genetics* [data availability policy](http://journals.plos.org/plosgenetics/s/data-availability), and numerical data that underlies graphs or summary statistics should be provided in spreadsheet form as supporting information.

Reviewer #1: Yes

Reviewer #2: Yes

Reviewer #3: Yes

PLOS authors have the option to publish the peer review history of their article ([what does this mean?](https://journals.plos.org/plosgenetics/s/editorial-and-peer-review-process#loc-peer-review-history)). If published, this will include your full peer review and any attached files.


If you choose “no”, your identity will remain anonymous but your review may still be made public.

**Do you want your identity to be public for this peer review?** For information about this choice, including consent withdrawal, please see our [Privacy Policy](https://www.plos.org/privacy-policy).

Reviewer #1: No

Reviewer #2: No

Reviewer #3: No

Responses to July 10, 2023 email from Nithya Venkatesan

Subject: PLOS Genetics Technical Check requests for your submission PGENETICS-D-23-00618R1 - [EMID:62e94d4d183b130a]

Dear Dr Bassler,

Thank you for submitting your paper, "Natural Silencing of Quorum-Sensing Activity Protects Vibrio parahaemolyticus from Lysis by an Autoinducer-Detecting Phage", to PLOS Genetics. We have checked over your submission and, before we can assign your manuscript to the Editor, we ask that you first please make the following modifications:

1) Please ensure that the CRediT author contributions listed for every co-author are completed accurately and in full. At this stage, "All Authors" require contributions. Please ensure that the full contributions of each author are acknowledged in the ""Add/Edit/Remove Authors"" section of our submission form.

The list of CRediT author contributions may be found here…

The contributions of each author are now acknowledged in the ""Add/Edit/Remove Authors"" section of our submission form.

2) Please ensure that the funders and grant numbers match between the Financial Disclosure field and the Funding Information tab in your submission form. Note that the funders must be provided in the same order in both places as well. Currently, order of grants are different in both places.

We used your online portal to add funders in the identical order as they appear in our manuscript file. However, we have had repeated difficulties with the auto-fill function on the portal. For that reason, we request that you transfer our funding information from our manuscript file into your system with the same names, numbers, and ordering as they appear in the manuscript text file.

3) In the online submission form you indicate that your data is available from a co-author on this manuscript.According to our Data Policy, data must either be included with the submission, be placed in a public repository, or available from a third party contact point who must not be an author on the manuscript. Please revise your data statement to a non-author institutional point of contact, such as a data access or ethics committee, and send this to us via return email. Please also include contact information for the third party organization (this contact should be a central address and not a named person), and please include the full citation of where the data can be found.

We apologize for the misunderstanding. All data are provided in the manuscript supporting information. The answer on the portal has been updated to reflect this.

4) Please provide an Author Summary. This should appear in your manuscript between the Abstract (if applicable) and the Introduction, and should be 150–200 words long. The aim should be to make your findings accessible to a wide audience that includes both scientists and non-scientists. Sample summaries can be found on our website under Submission Guidelines:

An author summary is now provided.

5) We notice that your supplementary Tables are included in the manuscript file.Please remove them and upload them  with the file type 'Supporting Information' leaving only the supplementary legends in the manuscript file. Please ensure that all Supporting Information files are included correctly and that each one has a Legend listed in the manuscript after the references list.

Done.

6) We have noticed that you have included SI Legends but few legends (S1 Data Points,S2 RNA-Seq data,S3 Raw Images) are missing.Please add a full list of legends for your Supporting Information file after the references list.

Done.

7) Some material included in your submission may be copyrighted. According to PLOS’s copyright policy, authors who use figures or other material (e.g., graphics, clipart, maps) from another author or copyright holder must demonstrate or obtain permission to publish this material under the Creative Commons Attribution 4.0 International (CC BY 4.0) License used by PLOS journals. Please closely review the details of PLOS’s copyright requirements here: PLOS Licenses and Copyright. If you need to request permissions from a copyright holder, you may use PLOS's Copyright Content Permission form.

Please respond directly to this email and provide any known details concerning your material's license terms and permissions required for reuse, even if you have not yet obtained copyright permissions or are unsure of your material's copyright compatibility. Once you have responded and addressed all other outstanding technical requirements, you may resubmit your manuscript within Editorial Manager.Potential Copyright Issues:

[Figure1(B)- Scissors]: Please confirm whether you drew the images / clip-art within the figure panels by hand. If you did not draw the images, please provide (a) a link to the source of the images or icons and their license / terms of use; or (b) written permission from the copyright holder to publish the images or icons under our CC BY 4.0 license. Alternatively, you may replace the images with open source alternatives. See these open source resources you may use to replace images / clip-art:

The source of the scissors image is Microsoft Office’s free icon collection. Per Microsoft policy: "These icons are free to use and there's no royalty or copyright to worry about." (<https://support.microsoft.com/en-gb/office/insert-icons-in-microsoft-365-e2459f17-3996-4795-996e-b9a13486fa79#:~:text=You%20can%20insert%20icons%20into,or%20copyright%20to%20worry%20about.>).

Thus, no further action is required as no license or permission is required.

Once you have made the necessary changes in Editorial Manager, please re-approve the submission by following the same steps as before.
